# Seismic Data Augmentation Based on Conditional Generative Adversarial Networks

**DOI:** 10.3390/s20236850

**Published:** 2020-11-30

**Authors:** Yuanming Li, Bonhwa Ku, Shou Zhang, Jae-Kwang Ahn, Hanseok Ko

**Affiliations:** 1 Department of Video Information Processing, Korea University, Seoul 136713, Korea; lym7499500@korea.ac.kr (Y.L.); bhku@ispl.korea.ac.kr (B.K.); 2 School of Electrical Engineering, Korea University, Seoul 02841, Korea; sujang@ispl.korea.ac.kr; 3 Korea Meteorological Administration, Seoul 07062, Korea; propjk@korea.kr

**Keywords:** generative adversarial networks, data augmentation, seismic waveforms

## Abstract

Realistic synthetic data can be useful for data augmentation when training deep learning models to improve seismological detection and classification performance. In recent years, various deep learning techniques have been successfully applied in modern seismology. Due to the performance of deep learning depends on a sufficient volume of data, the data augmentation technique as a data-space solution is widely utilized. In this paper, we propose a Generative Adversarial Networks (GANs) based model that uses conditional knowledge to generate high-quality seismic waveforms. Unlike the existing method of generating samples directly from noise, the proposed method generates synthetic samples based on the statistical characteristics of real seismic waveforms in embedding space. Moreover, a content loss is added to relate high-level features extracted by a pre-trained model to the objective function to enhance the quality of the synthetic data. The classification accuracy is increased from 96.84% to 97.92% after mixing a certain amount of synthetic seismic waveforms, and results of the quality of seismic characteristics derived from the representative experiment show that the proposed model provides an effective structure for generating high-quality synthetic seismic waveforms. Thus, the proposed model is experimentally validated as a promising approach to realistic high-quality seismic waveform data augmentation.

## 1. Introduction

In the past hundred years alone, earthquakes have brought many disasters upon people. Earthquakes have become one of the most important issues for the growing world population and driven scientists and engineers to study them [1]. As a result, accurate and quick automated earthquake detection is a high priority across the world. The recent development of automated earthquake detection and classification has seen various seismic signal processing techniques proposed [2,3,4]. In particular, machine learning incorporating deep learning has been receiving great attention in statistical seismology. Deep learning algorithms, such as convolutional neural networks (CNNs), have been successful in image classification [5,6] and recurrent neural networks (RNNs) have achieved promising results with time series data [7,8]. Since one of the main advantages of deep learning is its ability to automatically extract suitable features from massive amounts of seismic data from a variety of seismic sensors, deep learning-based algorithms can solve seismic problems more accurately than traditional methods. Deep CNN architectures were used for earthquake detection and rough localization, and they demonstrated more computational efficiency with higher accuracy than some prominent traditional algorithms [9]. More recently, deep learning algorithms combined CNNs and RNNs to automatically pick P/S-wave arrival times, achieving significant improvements over the traditional method [10].

Deep learning models using only a small dataset tend to overfit during their training process. To solve this problem, data augmentation techniques have proved effective at enhancing dataset volume and quality. In seismology, the data augmentations principally used involve adding background noise, permutation, and cropping [11]. However, while such augmentation techniques increase dataset quantity, dataset quality is lacking. A generative adversarial network (GAN) is an artificial intelligence algorithm used for unsupervised learning that can be applied to data augmentation. The main idea of GANs is to generate fake samples from a simple distribution like Gaussian noise and learn to map the noise distribution to the data distribution by an adversarial training of two networks [12]. GANs are used to generate not only audio and visual data but also seismic wave data [13,14,15,16,17,18]. Although previous GANs have primarily been focused on generating short-term seismic waveforms, in seismology long-term seismic waveforms containing P and S-wave information are also important.

In this paper, we propose a conditional GANs based model to generate synthetic seismic waveforms for data augmentation. The conditional GANs learn to generate the synthetic samples from a specific prior or set of characteristics rather than just random Gaussian noise. The specific prior can be an image, a label associated with classes, or a more detailed tag. Our model is composed of three parts: generator, discriminator and pre-trained feature extractor, and the main contributions are as follows.

(1) We propose a novel seismic waveform generating model based on a conditional GAN-based network. Instead of using random noise as the input to the generator, the embedding statistics of real seismic waveforms are used as inputs.

(2) In addition to the adversarial loss of GANs, we add a content loss related to high-level information to the objective function to increase the quality of the synthetic data. To achieve this, we apply a pre-trained feature extractor to our architecture.

We then evaluate the quality of the synthetic data by seismic waveforms analysis and classification performance analysis. Representative experimental results demonstrate that our model is a promising approach to augmenting seismic data.

## 2. Background

### 2.1. GANs

Generative adversarial networks (GANs) were proposed by Goodfellow a few years ago [12]. They are able to learn how to generate real data distributions by training two competing neural networks: a generator and a discriminator (or critic). The aim of the generator is to capture the distribution of real data for fake data generation. In other words, the generator needs to generate fake data as realistically as possible. Meanwhile, the role of the discriminator is to distinguish between fake and real data. The training progresses based on an adversarial min-max game between the two networks. The optimization of the GANs terminates when the discriminator is no longer able to distinguish the real data from the synthetically generated data.

**Figure 1:**
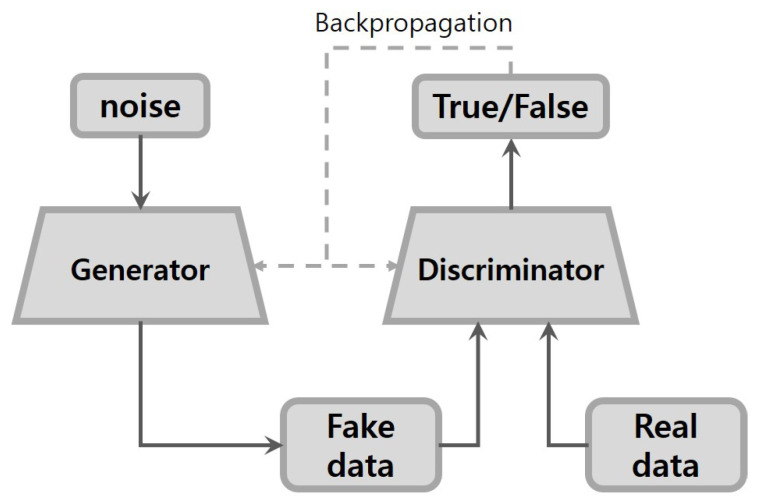
Architecture of basic generative adversarial networks (GANs).

The basic GANs framework is presented in Figure 1. The input to the generator is Gaussian noise, and the output is fake data, which, along with the real data, would be labeled as true or false by the discriminator. The GANs model can be trained by optimizing the objective function as follows:

(1) $$\min_{G}\max_{D}V\left(D,G\right)=E_{x∼P_{data(x)}}\left[\log D(x)\right]+E_{z∼P_{z}}\left[\log\left(1-D(G(z)\right)\right]$$

The function above is simply the binary cross-entropy cost function. D(x) is the probability of real data *x* estimated by discriminator. G(z) is the fake data generated by a generator when given noise *z*. D(G(z)) is the probability of fake data G(z) estimated by discriminator. When distinguishing between the real and the synthetic data, the discriminator learns to maximize both the log of the predicted probability of the real data and the log of the inverse of the predicted probability of the fake data. Conversely, the generator tries to minimize the log of the inverse of the predicted probability of the fake data. However, the generation of high-resolution images has proven difficult, particularly i-th high variability datasets [19].

### 2.2. Conditional GANs

**Figure 2:**
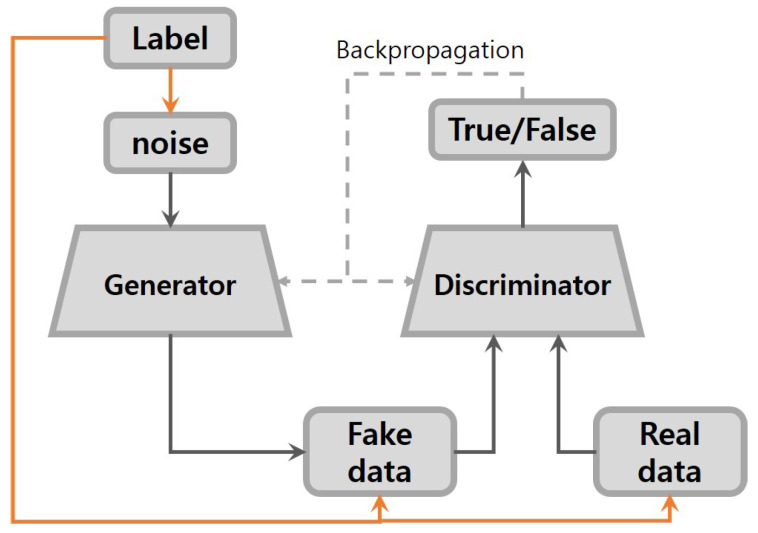
Architecture of conditional GANs.

As shown in Figure 2, the conditional GANs are similar to basic GANs except for some extra information that is imposed on both the generator and discriminator inputs [20]. The generator learns a representation of noise that is independent of the conditional information. By incorporating this condition into the objective function of basic GANs, the objective function of conditional GANs becomes as follows:

(2) $$\min_{G}\max_{D}V\left(D,G\right)=E_{x∼P_{data(x)}}\left[\log D(x|y)\right]+E_{z∼P_{z}}\left[\log\left(1-D(G(z|y)\right)\right]$$

where *y* is the extra information of the real data, whether labels of classes or images. D(x|y) and D(G(z|y)) are the probability of real data *x* and fake data G(z) conditioned on *y* estimated by discriminator, respectively. The conditioning is performed by feeding y into both the discriminator and generator as an additional input layer.

If the extra information is utilized well, the quality of the generated data is dramatically increased. There have been many variants of conditional GANs. For example, the Auxiliary Classifier GAN (ACGAN) builds upon conditional GANs [21]. In terms of the generator model, the ACGAN, like conditional GANs, is provided with both noise and class labels as inputs. The main difference from conditional GANs is in the discriminator, in which the input is only unlabeled data. The output predicts whether the given data are real or fake, and predicts the class label. The modification of conditional GANs in this way causes the training process to be more stable and have an improved ability to generate larger images.

Another successful conditional GANs based model is pix2pix, which is used for image-to-image translation [22]. Unlike prior works, the pix2pix uses U-Net as a generator. The GANs learn a mapping from the distribution of Gaussian noise to the distribution of real data. In pix2pix, the input to the generator is image data rather than noise. The generator of pix2pix learns to generate plausible images in the target domain from the images in another domain. Another contribution of this model is that the generator is updated via the L1 loss obtained between the generated image and the expected output image. This additional loss enhances the generator to create plausible translations of the source image. The objective function of pix2pix can be expressed as:

(3) $$\begin{array}{cc} L_{adv}\left(D,G\right) & =E_{x∼P_{data(x)},y∼P_{y}}\left[\log D(x,y)\right]+E_{y∼P_{y}}\left[\log\left(1-D(x,G(x,z)\right)\right]\\ L_{l_{1}}\left(G\right) & =E_{x∼P_{data(x)},y∼P_{y}}{[∥y-G\left(x,z\right)∥}_{1}]\\ L_{pix2pix} & =L_{adv}+\lambda L_{l_{1}} \end{array}$$

where λ is a hyper parameter and *y* is the conditional images used as inputs to the generator. D(x,y) is the probability of input image *x* conditioned on *y* estimated by discriminator. D(x,G(x,z)) is the probability of fake image G(x,z) conditioned on *y* estimated by a discriminator. Additionally, the L1 distance between the generated images and the ground truth is added to the objective function. This helps to make generated images closer to the ground truth.

## 3. Seismic Signal Synthesis with Conditional GANs

**Figure 3:**
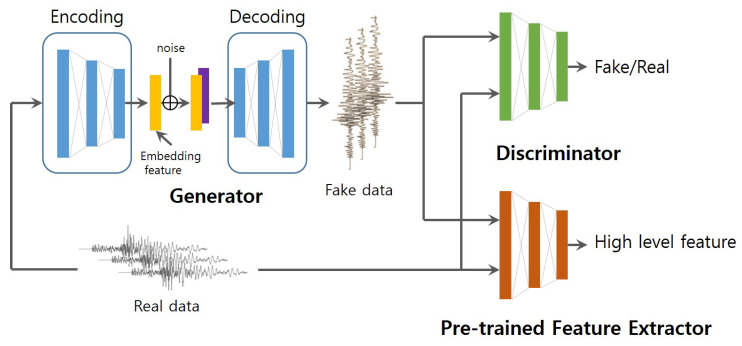
Architecture of proposed model.

The proposed model is composed of three parts, as shown in Figure 3: (1) generator, (2) discriminator, and (3) pre-trained classifier. In our task, we want the generated seismic signals to retain the important characteristics of real seismic signals, such as P/S-wave arrival times and amplitudes. The generator model consisting of an encoder and a decoder generates fake samples, while the discriminator model distinguishes them based on authenticity. To further improve the perceptual quality of the fake data, a pre-trained classifier is trained by real events and noise added into the network. The pre-trained classifier does not participate in the optimization during training.

### 3.1. Network Architecture

Compared to the fake data created by generators learning to map real data from only Gaussian noise, fake data conditioned by real data can generate realistic data for data augmentation. The architecture of our proposed model, which is based on pix2pix, is presented in Figure 3. The generator model generates fake data samples, while the discriminator model distinguishes them for authenticity. To further improve the perceptual quality of the fake data, a pre-trained classifier that is trained by real events and noise is added in the network. The pre-trained classifier does not participate in the optimization during training but extracts the high-level features from the fake and real data and minimizes the L2 distance between them.

#### 3.1.1. Generator

**Figure 4:**
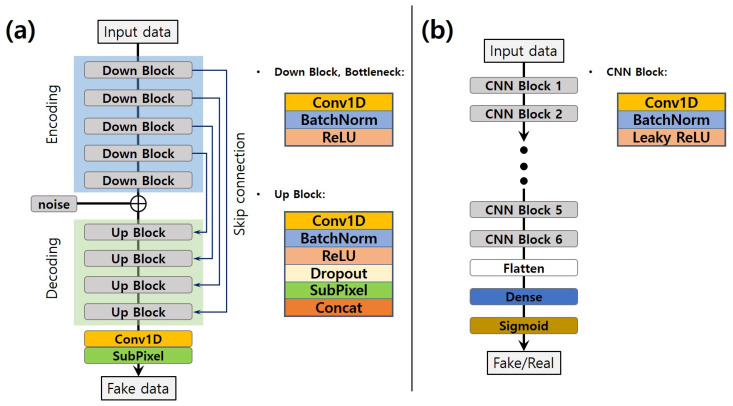
Architecture of (**a**) generator and (**b**) discriminator.

The generator model is composed of an encoder-decoder structure as shown in Figure 4a. The encoder maps the input data into a compressed latent space through a down-sampling, while the decoder reconstructs the compressed latent features to the original space through an up-sampling. In order to prevent the information loss that can occur in encoder-decoder structures, we use U-Net with a skip-connection.

The input and output data of the generator contains three channels of 1D time series data of length 2048. The encoding layer is composed of four down-blocks, the decoding layer is composed of four up-blocks, and a bottleneck layer lies in between. The down-block and bottleneck perform a 1-D convolution, batch normalization, and ReLU non-linearity function. The down-block d=1,2,3,4,5 contains c=16,32,64,128,128 filters with the kernel size k=5,5,5,3,3 of stride s=2. The feature maps of the encoding module are concatenated with Gaussian noise before input to the decoding module. The up-blocks are similar to the down- blocks. However, Subpixel, which is an up-sampling technique, is added to the up-blocks [23] to solve the problem of checkerboard artifacts in the decoding process. The up-block u=1,2,3,4 contains c=128,64,32,16 filters with the kernel size k=3,5,5,5 of stride s=1. Additionally, when the input and output are similar, the features of down-sampling can aid up-sampling [22]. Thus, the correspondingly-sized down-sampling features and up-sampling features are concatenated as inputs to each successive up-sampling layer. Finally, the last up-sampling is through the combination of a 1-D convolution and Subpixel layers.

#### 3.1.2. Discriminator

As shown in Figure 4b, the discriminator model contains six CNN blocks and a fully connected layer (also called a dense layer). The discriminator takes the same size of input data as the generator and outputs a scalar value indicating whether the data are real or fake (real:1, fake:0). The CNN block is composed of 1-D convolution, batch normalization, and a leaky ReLU non-linearity function. Each convolutional layer has 64 filters, a kernel size k=10, and stride s=2. The fully connected layer that follows the output of CNN blocks has 2048 neurons.

#### 3.1.3. Pre-Trained Feature Extractor

**Figure 5:**
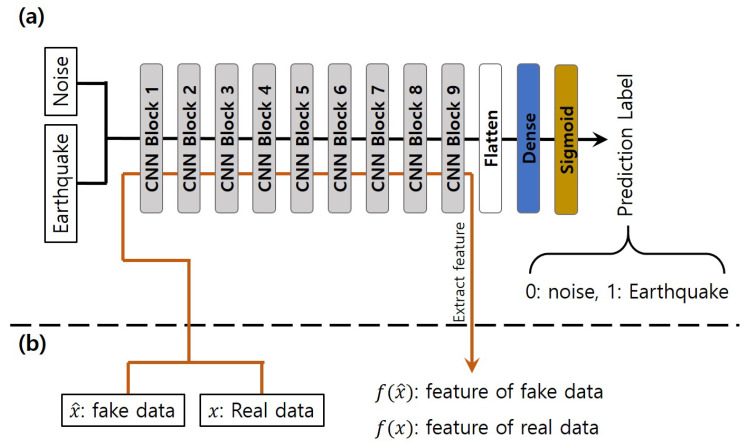
(**a**) Architecture of pre-trained classifier, (**b**) the schematic diagram of pre-trained classifier employed in GAN training.

The architecture of the pre-trained feature extractor is similar to the discriminator. To further extract the high-level features of the input data, we stack nine CNN blocks, as shown in Figure 5a. Each convolutional layer has 64 filters, a kernel size k=10, and stride s=2. The pre-trained feature extractor and the discriminator use the same CNN blocks. We trained the feature extractor to distinguish between earthquakes and noise. When training the GANs, the pre-trained classifier is used to extract the high-level features, which are the outpour of the last CNN block, and the pre-trained feature extractor does not contain the optimization process of the GANs.

### 3.2. Loss Function

The loss function of GANs can be expressed as

(4) $$\min_{G}\max_{D}V\left(D,G\right)=E_{x∼P_{data(x)}}\left[\log D(x)\right]+E_{x∼P_{data(x)},z∼P_{z}}\left[\log\left(1-D(G(x,z)\right)\right]$$

where *G* and *D* are the generator and discriminator, respectively. The generator tries to fool a differentiable discriminator. Meanwhile, the discriminator tries to distinguish the fake data G(x,z) (reconstructed seismic) from real data *x*. The *G* is trained to minimize this objective against an adversarial *D* that is trained to maximize it.

The content loss is utilized to retain the consistency of high-level features between the real and fake data, and we build on the idea of using a loss function that reduces the distance between the fake and real data in perceptual space [16]. The content loss is the mean square error between the two sets of feature maps as follows:

(5) $$L_{content}\left(G\right)=E_{x∼P_{data(x)},z∼P_{z}}{[∥f\left(x\right)-f\left(G\left(x,z\right)\right)∥}_{2}]$$

where f(.) is the pre-trained feature extractor, f(x) and f(G(x,z)) are feature maps from real data and fake data, respectively.

Our final loss function is written with trade-off parameter λ

(6) $$L_{full}=L_{adv}+\lambda L_{content}$$

## 4. Experiment and Analysis of Results

### 4.1. Training Details and Data Preprocessing

In this paper, the experiment was implemented on an NVIDIA GeForce RTX 2080 Ti, a Windows 10 operating system, and the simulation software is written in Python. We used a publicly available earthquake dataset, the STandford Earthquake Dataset (STEAD), to test our model [24]. This dataset is a global dataset of earthquake and non-earthquake (noise) of high-quality and large scale. We selectively used earthquake events of magnitude greater than 3.0. The total number of earthquake events and noise samples are 34,569 and 28,211, respectively. The seismic signals are recorded at a sample rate of 100 Hz on three channels. For optimization, we used the ADAM algorithm with the learning rate set to $10^{-5}$. In the GAN training process, the batch size and epoch were set to 64 and 1000, respectively.

**Figure 6:**
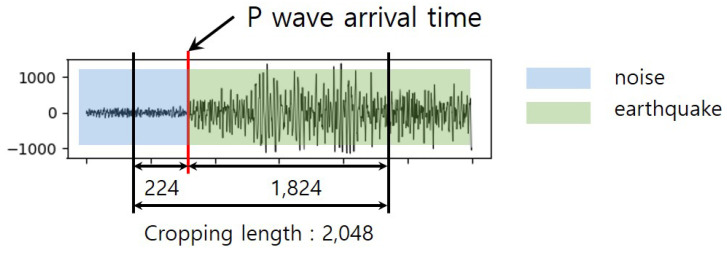
Schematic diagram of seismic signal cropping with 2048 window size.

For training the GANs, we just used the earthquake events dataset. The training and test dataset is divided according to a 7:3 ratio, making 24,198 and 10,371 samples, respectively. In order to present the phase of the P wave, as shown in Figure 6, we cropped the data starting 224 before the P wave arrival time with a 2048-long window. In addition to this, we applied a high-pass filter to mitigate the effect of noise on the seismic signals and pass the signals with a frequency greater than 1 Hz [25]. Carrying out a min-max normalization for each input to rescale the range of the amplitude to scale the range within [0,1],

(7) $$y=\frac{x-min(x)}{max(x)-min(x)}$$

where *x* is the original value, min(x) and max(x) are minimum and maximum value of *x*, respectively.

Regarding the generated seismic signals, the values at the endpoint of the time series data usually contain anomalous data. We crop the points by a length of 24 at both ends of the generated seismic data to obtain the final seismic signals of the length of 2000.

### 4.2. Results and Discussion

#### 4.2.1. Analysis Results by Visual Comparison

**Figure 7:**
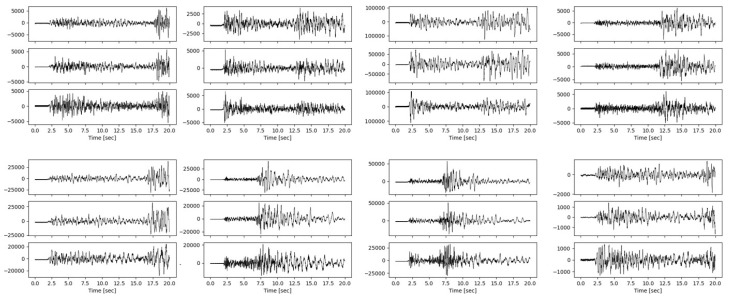
Samples of synthetic seismic waveforms generated by our model.

Traditional earthquake detection depends on the appearance of seismic waveforms. Thus, the visual realism of the synthetic waveform is an important metric. The trained generator has gained the ability to produce realistic synthetic waveforms by mapping random noise conditioned by embedding features of real seismic waves. Figure 7 shows examples of created synthetic seismic waveforms. We observe that the synthetic seismic waveforms show several essential features similar to those of real P waveforms and S waveforms, including clear onsets of P waves and S waves, the signal-to-noise ratio, and a part of the noise signal at the beginning. This indicates that the generator has actually learned the statistical characteristics of the real earthquake dataset. Although the input to the generator is real seismic data, note that the synthetic samples are neither noisy versions nor copies of the real data. The generator does not have a complete reconstruction of the real data during the training process but generates the synthetics on the basis of ensuring the important features of real input data. These synthetic waveforms generated from the concatenation of embedded features and random noise are more realistic than the synthetic waveforms generated from random noise only. Additionally, GAN architecture with conditional information can readily generate large volumes of data. Thus, the synthetic data created could aid seismic data augmentation in deep learning for seismology.

**Figure 8:**
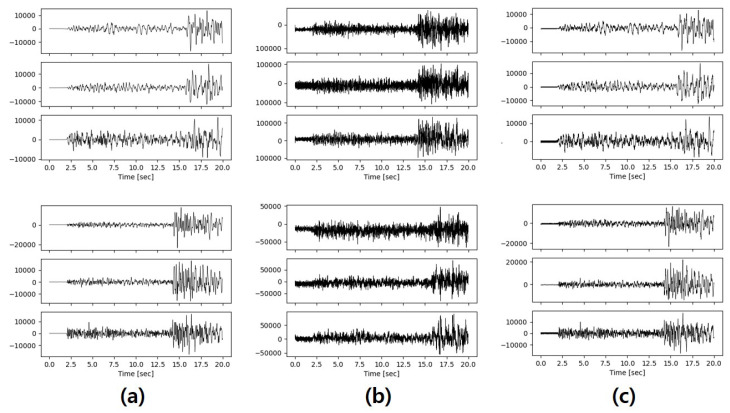
(**a**) Shows the sample’s input to the generator, (**b**) shows samples generated by models without the pre-trained feature extractor, and (**c**) shows the samples generated by our model.

To validate the importance of the pre-trained feature extractor in our model, we conducted visual evaluations comparing the performance of the model with and without it using the same generator and discriminator. A comparison of samples from the inputs and outputs of the generator models are shown in Figure 8. The input and output data correspond to the real seismic data and synthetic seismic data, respectively. The S wave arrivals of the synthetic samples from both models are clear on all three channels. However, the p wave arrivals are not apparent in the synthetic samples generated by the model without the feature extractor. However, in the synthetic samples generated by our model, the P wave arrival is well synthesized. These results show that the feature extractor can help the generator retain some important characteristics in the process of reconstructing the input seismic data and fully validate the role of feature extractor within the whole framework.

**Figure 9:**
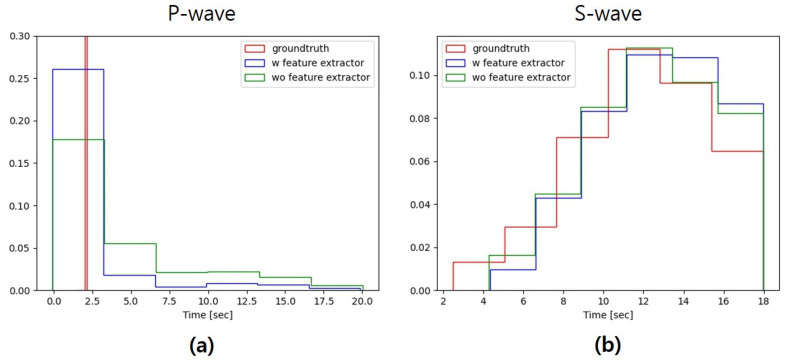
Comparisons of the picked arrival times distribution in the testing dataset. The blue and green histograms indicate the distributions of P- and S- wave arrival times in the synthetic seismic data generated by the architecture with or without feature extractor, respectively. The red histogram indicates those distributions in real data. (**a**) P-wave and (**b**) S-wave.

We also analyzed the distribution of the picked arrival times. We used an AR-AIC +STA/LTA algorithm to pick P and S arrival times. Since the details of the P and S arrival times of the real data are provided, only the synthetic seismic data requires picking arrival times. We compared the three datasets as follows: the real dataset, the synthetic dataset generated by our model, and the synthetic dataset generated by our model without the feature extractor. As shown in Figure 9a, the P arrival times of the real data are 2 s, and most of the synthetic P arrival times are also clustered around 2 s. More importantly, the P arrival time distributions of the real data and synthetic data created by our model are more comparable than the other synthetic data. Comparing the S arrival time distribution in Figure 9b, revere a high degree of similarity between all the datasets. Generating P waves is more difficult than generating S waves because, in general, the amplitude difference between the P waves and the noise is much smaller than the amplitude difference between the S waves and the P waves. Thus, the noise portion and P wave portion of generated data is more difficult to distinguish. Additional constraint loss calculated on the feature map allows the realism of the noise and P waves to be enhanced.

#### 4.2.2. Time-Frequency Domain Analysis

**Figure 10:**
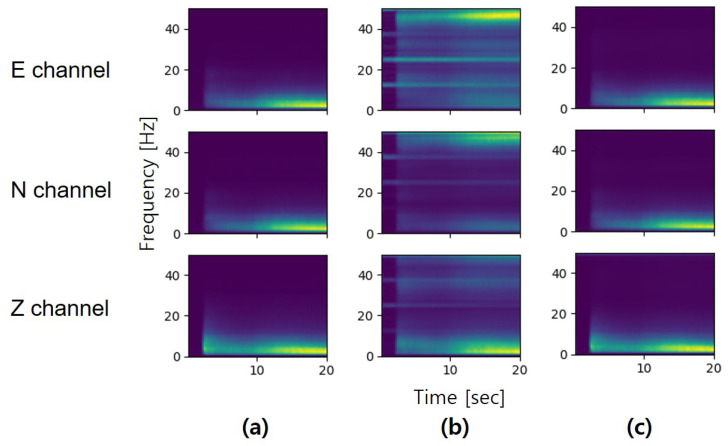
Mean of spectrogram obtained by: (**a**) real dataset, (**b**) synthetic dataset (w/o feature extractor) and (**c**) synthetic dataset (with feature extractor).

**Figure 11:**
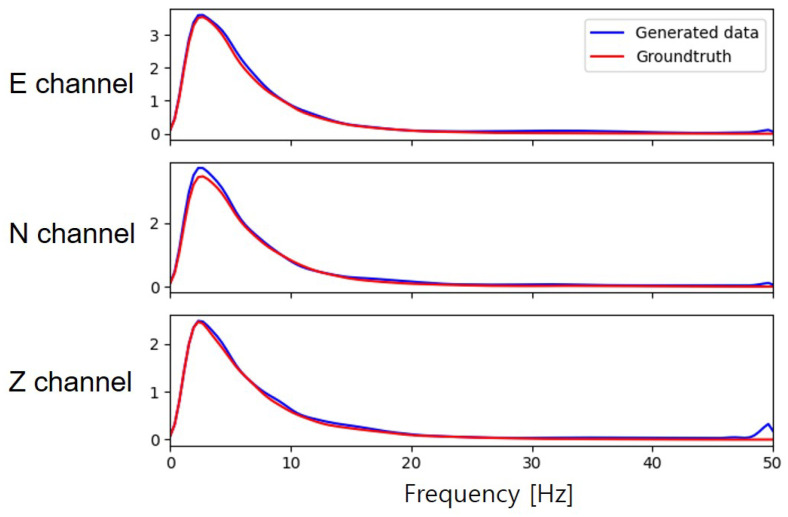
The spectrum comparison between the real dataset and synthetic dataset.

To further verify the quality of the synthetic seismic data, we also evaluate the synthetic data in the time-frequency domain. We first obtained the spectrograms by computing the Short-Time Fourier Transform (STFT) on the seismic waveforms and then calculated the mean of spectrograms for each dataset. Figure 10 demonstrates that our model can successfully generate realistic seismic data. Comparing the mean spectrograms, the synthetic seismic data has the same characteristics in the time-frequency domain. Meanwhile, the results also show the importance of the feature extractor in our architecture. The feature extractor can enhance the components of the seismic signals and reduce the generation of unnecessary noise. In addition, we compared the mean of the spectrum between the real dataset and synthetic dataset as shown in Figure 11. Across most of the frequency range, the frequency components exhibit a high degree of similarity between the real dataset and synthetic dataset. However, the limitation of our model is that the synthetic seismic waveforms contain noise with a frequency greater than 48 Hz, especially in the z-channel. The bandpass filter can be used to reduce this effect.

#### 4.2.3. Analysis Results by Classification

In another evaluation method, we assess the quality of synthetic seismic samples by using a classification model that is trained with the augmented dataset. The classification model is based on ‘ConvQuakeNet’ [9]. Our classifier is composed of nine 1D convolutional layers and a fully connected layer. Additionally, we use batch normalization after the convolutional layer to stabilize the model during the training process. We use the learning rate of $10^{-5}$ for training the classification model. The batch size and training epoch is set to 128 and 100, respectively. We divide the dataset into a real seismic training and test sets size of 10,371 and 5000, real noise training and test sets size of 19,747 and 8464. The size of the generated synthetic seismic dataset is 10,371. We employ the accuracy, recall, precision, and F1 score to evaluate the performance. The definitions of these metrics as follows:

(8) $$Accuracy=\frac{TruePositive+TrueNegative}{Total}$$

(9) $$Recall=\frac{TruePositive}{TruePositive+FalseNegative}$$

(10) $$Precision=\frac{TruePositive}{TruePositive+FalsePositive}$$

(11) $$F1\;score=2*\frac{Precision*Recall}{Precision+Recall}$$

Several datasets were used to training the classification model. In this experiment, the seismic and noise training set size is fixed. Then, the seismic training set is augmented with different sizes of synthetic data. We increased the size of the seismic training set by synthetic seismic data with a size of 20%, 40%, 60%, 80%, and 100% number of real seismic data. In addition to this, we also employed a seismic training set containing only synthetic data. As shown in Table 1, the performance of the classification using a real dataset is better than the one trained on a synthetic dataset. With the different sizes of data augmentation, the results show additional synthetic data may cause performance degradation. However, selecting a suitable amount of synthetic data will increase the classification performance. When we add 60% of synthetic data into the real dataset, the accuracy is increased from 96.84% to 97.92%. Although the precision is slightly reduced, the recall has a robust improvement. The result of the F1 score also indicates data augmentation with synthetic data can improve the performance of classification.

## 5. Conclusions

In this paper, we proposed a GANs-based generative model that can produce synthetic seismic waveforms. Our model is based on a conditional GAN augmented with a third model to enhance the quality of the generated seismic waveforms. To verify the performance of the generative model, we analyzed the synthetic seismic waveforms in both the time domain and the time-frequency domain. Experimental results show that the proposed model can generate high-quality synthetic waveforms that realistically incorporate the characteristics of real data, including retaining the noise portion. In addition, the classification results were shown to prove that accuracy is increased with the proposed data augmentation. The proposed generative model can be widely applied to many potential usage scenarios in seismology. As future work, we will investigate a generative model that can produce longer-term synthetic seismic waveforms.

## Figures and Tables

**Table 1:** The classification results of the classifier trained with real data, synthetic data, and real data augmented by different synthetic data size.

Dataset	Accuracy	Precision	Recall	F1 Score
Real	96.84	99.96	91.54	0.956
Synthetic	90.45	97.86	75.94	0.855
Real + 20%	94.60	99.93	91.04	0.953
Real + 40%	95.65	99.95	88.32	0.938
Real + 60%	97.92	98.74	95.62	0.972
Real + 80%	95.55	96.03	91.82	0.939
Real + 100%	94.80	88.28	99.14	0.934
